# Dynamic gait phase alterations and spatiotemporal stance characteristics in bilateral pes cavus: A case-control study

**DOI:** 10.1371/journal.pone.0359862

**Published:** 2026-10-06

**Authors:** Lucía Martínez-Salvador, Helena Silva-Migueis, Ricardo Becerro-de-Bengoa-Vallejo, Marta Elena Losa-Iglesias, Javier Bayod-López, Israel Casado-Hernández, Juan Gómez-Salgado, Daniel López-López

**Affiliations:** 1 Research, Health and Podiatry Group, Department of Health Sciences, Faculty of Nursing and Podiatry, Industrial Campus of Ferrol, Universidade da Coruña, Ferrol, Spain; 2 CrossI&D Lisboa Research Center, Escola Superior de Saúde da Cruz Vermelha Portuguesa, Lisboa, Portugal; 3 Faculy of Nursing, Physiotherapy and Podiatry, University Complutense de Madrid, Madrid, Spain; 4 Faculty of Health Sciences, Universidad Rey Juan Carlos, Madrid, Spain; 5 Applied Mechanics and Bioengineering Group (AMB), Aragon Institute of Engineering Research (I3A), Universidad de Zaragoza, Zaragoza, Spain; 6 Department of Medicine and Public Health, Faculty of Labour Sciences, University of Huelva, Huelva, Spain; 7 Safety and Health Postgraduate Programme, Universidad Espíritu Santo, Guayaquil, Ecuador; University of Szeged Institute of Biology: Szegedi Tudomanyegyetem Biologia Intezet, HUNGARY

## Abstract

**Background:**

Dynamic baropodometric assessment is an established technique for evaluating functional foot biomechanics and gait alterations associated with cavus foot deformities. The primary objective of this study was to analyze spatiotemporal gait variables and stance phase progression in individuals with bilateral pes cavus compared to healthy control participants with normal foot posture.

**Methods:**

A total of 82 participants were enrolled in this observational case-control study, comprising 41 individuals with bilateral pes cavus (case group) and 41 healthy individuals with normal bilateral foot posture (control group). Following the validated three-step protocol, participants walked barefoot across a calibrated baropodometric platform. Spatiotemporal parameters during the stance phase were recorded, including absolute duration (ms) and stance percentage (%) for the Initial Contact Phase (ICP), Flatfoot Phase (FFP), and Forefoot Contact Phase (FFCP), along with their corresponding transition frame indices. Group comparisons were performed using the Mann-Whitney U test.

**Results:**

In the right foot, participants with pes cavus exhibited a significantly longer ICP duration (109.29 ± 63.10 ms vs. 79.71 ± 35.35 ms, p = 0.034) and higher ICP stance percentage (14.46 ± 8.27% vs. 10.20 ± 4.55%, p = 0.024), accompanied by a markedly reduced FFP duration (347.95 ± 127.31 ms vs. 417.20 ± 92.98 ms, p < 0.001) and lower FFP percentage (47.68 ± 14.05% vs. 56.02 ± 10.45%, p = 0.002). In the left foot, the case group demonstrated a reduced FFP duration (341.61 ± 115.79 ms vs. 405.73 ± 72.40 ms, p = 0.002) and a significantly higher FFCP percentage (37.05 ± 8.28% vs. 32.71 ± 7.85%, p = 0.020), alongside lower FFCP frame transition indices p = 0.002.

**Conclusions:**

Individuals with bilateral pes cavus present significant alterations in dynamic stance phase progression, characterized by prolonged initial contact, restricted midfoot flatfoot support duration, and premature transition toward forefoot contact. These findings highlight altered dynamic gait mechanics in cavus feet and support the clinical utility of baropodometric gait analysis for functional assessment and orthotic planning.

## Introduction

Pes cavus (PC), or cavovarus foot deformity, is a complex structural condition characterized by fixed or flexible plantar flexion of the forefoot relative to the hindfoot, resulting in an abnormally elevated longitudinal arch [1]. In addition to sagittal plane misalignment, cavovarus feet are typically defined by structural rigidity and reduced soft tissue compliance, contrasting with flatfoot deformities that exhibit lower arches and joint hypermobility [2]. Population-based estimates regarding the prevalence of pes cavus vary depending on diagnostic criteria, ranging from approximately 10% in general cohorts [3,4] to as high as 25% among specialized patient groups, such as individuals with diabetic neuropathy [5]. Studies indicate no significant disparity in prevalence between biological sexes [6].

The biomechanical consequences of pes cavus tend to worsen over time if left unmanaged. Although clinical presentation often becomes pronounced during adolescence or early adulthood, symptoms can present across all age groups [7]. Early identification and intervention are essential to prevent secondary musculoskeletal complications; however, high-level clinical evidence regarding conservative and surgical management options remains limited [7].

Regarding etiology, unilateral pes cavus frequently stems from traumatic injuries, whereas bilateral deformities are predominantly associated with hereditary or neurological conditions [8]. Neuromuscular imbalances, particularly in Charcot-Marie-Tooth disease, represent a common underlying cause of progressive cavovarus deformities [9].

In clinical practice, static assessment tools such as Coleman’s block test, the Foot Posture Index, and the Navicular Drop Test (NDT) are routinely utilized to evaluate foot posture and flexibility [10–12].

Patients with pes cavus frequently report localized discomfort, callosities, and altered gait mechanics [13].

Functional assessment of gait is therefore critical to evaluate dynamic stance characteristics. The center of pressure in a functional foot transitions smoothly from heel strike through midfoot stance to forefoot push-off. Portable baropodometric platforms provide a practical, reliable method to capture dynamic spatiotemporal parameters during gait stance [14]. Although there are some research on the subject, there is no comprehensive database from which to obtain information on the characteristics of the PC while walking over a pressure platform [7].

Although dynamic stance alterations in cavus feet have been recognized, detailed quantification of spatiotemporal phase transitions remains underreported. The primary objective of this study was to compare dynamic gait phase durations and transition metrics between individuals with bilateral pes cavus and healthy controls. We hypothesized that participants with bilateral pes cavus would display a prolonged Initial Contact Phase (ICP) due to impaired calcaneal shock absorption, a shortened Flatfoot Phase (FFP) reflecting structural midfoot rigidity, and an accelerated transition into the Forefoot Contact Phase (FFCP).

## Material and methods

### Study design

A descriptive, observational case-control study was conducted between November 11, 2024 and December 20, 2024. A consecutive, non-random technique was used to recruit a total sample of 82 participants (11 men and 71 women), aged from 18 through 64 years old. The Bioethics Committee of University of A Coruña (Spain) approved the research on October 2024 with ID: 2024−033. This study followed the *S*trengthening the Reporting of Observational Studies in Epidemiology (STROBE) guidelines [15]. Moreover, it was in line with the Declaration of Helsinki and Organic Law 3/2018, of 5 December, on the protection of personal data and the correct guarantee of digital rights. This enabled the investigation to follow current regulations on human [16].

### Inclusion and exclusion criteria

The inclusion criteria were: 1) Subjects with an age between 18 and 65 years old, 2) Clinical confirmation of bilateral pes cavus for the case group, or bilateral normal foot posture for the control group, validated via the Navicular Drop Test (NDT) 3) Absence of lower limb surgical history or major trauma; and 4) Signed written informed consent.

The exclusion criteria were: 1) Lower extremity malformations, structural lower limb length discrepancies, or prior orthopedic surgery 2) Diagnosed neuromuscular, systemic vascular, or metabolic disorders altering gait; and 3) Active pregnancy at the time of evaluation.

### Sample size

The sample size was determined using Epidat 4.2 software (Consellería de Sanidade, Xunta de Galicia, Spain). The sample size calculation was based on pilot data evaluating the temporal variances of the Flatfoot Phase (FFP) duration. Assuming a medium-to-large effect size (*Cohen’s d = 0.65*) and an expected standard deviation (SD) of ± 85ms, a two-tailed test was configured with an 80% statistical power (*type II error = 20%)* and a 5% significance level (*type I error = 5%).* The mathematical analysis indicated a minimum recommendation of 38 participants per group. To compensate for any potential losses during dynamic recording or subsequent data processing, a final cohort of 82 individuals was recruited (41 participants per group).

Participants for both cohorts were recruited via convenience sampling at the gait and movement analysis laboratory of the University of A Coruña, comprising university students, staff, and associated family members who met the eligibility criteria. The control group was selected to match the case group’s age and sex profile. Control subjects were successfully included only after a senior clinician confirmed a completely healthy, asymptomatic musculoskeletal status and verified a neutral, normal bilateral foot posture, objectively defined by a Navicular Drop Test (NDT) displacement strictly within the physiological range of 5–9 mm.

## Methods

A senior trained podiatrist with more than 10 years of clinical experience carried out baseline measurements throughout four consecutive phases:

During the first phase it was conducted a structured interview. It consisted of several questions related to demographic data and relevant medical background, as history of personal diseases, injuries, health status, sex and age, and predictors for possible illnesses that could change the gait pattern, for instance, diabetes mellitus, vascular alterations or sedentary behaviour.

Regarding the second phase, anthropometric measurements were completed using the Quetelet’s equation (*BMI = Weight / Height*^*2*^) [17]. All weight (kg) and height (m) records were obtained using standardized digital scales and stadiometers prior to clinical evaluation.

During the third phase, the podiatrist carried out a full inspection of both feet of all individuals. The aim was to observe and evaluate the musculoskeletal mobility they possessed, muscle strength, imbalances, and structural integrity according to the *Kendall* manual muscle test [18]. To evaluate the intrinsic and extrinsic foot muscles, the subjects carried out with active and passive movements of foot plantarflexion, dorsiflexion, inversion and eversion. Manual resistance was applied to every movement and compared with the contralateral lower limb too. The scale employed six grades, going from 0, or no palpable nor visible muscle contraction, to 5, or complete range of motion against gravity with maximal resistance.

Lastly, the fourth phase carried the foot classification into flat, normal or healthy, and cavus types. The Navicular Drop Test (NDT) was performed to assess the mobility of the foot, to observe, and to quantify in millimetres the displacement of the scaphoid bone in the sagittal plane. The congruency of the subtalar joint of the foot is palpated while the patient is asked to sit with both legs flexed with the hip and knee at 90° to the ground. The navicular tuberosity is then located and marked and the distance to the ground is determined with a ruler. Finally, the patient is told to stand up with both feet at the same time, and the position of the navicular is reassessed, marked, and the distance to the ground is again measured. The whole procedure was repeated 3 times and then the arithmetic mean of the results was calculated [12]. The NDT has a good reliability for foot assessments [19].

In addition to this measurement, a portable baropodometric platform with 1600 autocalibrated resistive sensors was used to study the plantar loading distribution. The Neo-Plate (Herbitas, Spain) software was applied as it is a validated device for foot diagnosis [20]. The platform operates at a uniform data acquisition frequency of 100 Hz to 150 Hz. with a surface of 565 mm length x 612 mm width x 22 m height and an analysis area of 400 mm x 400 mm. Each sensor was 8 mm thick with 10 mm x 10 mm distribution.

Participants completed the validated “three-step protocol” walking barefoot at a self-selected natural velocity [21,22]. Participants walked barefoot at a self-selected, comfortable pace, ensuring the target foot made contact during the third step of the gait cycle. Four valid trials were recorded per side, and average values were used for statistical analyses. The stance phase was divided into three sequential phases: Firstly, Initial Contact Phase (ICP): From initial heel strike to early plantar contact onset; Secondly, Flatfoot Phase (FFP): Period of total plantar foot contact and thirdly, Forefoot Contact Phase (FFCP): Phase from heel elevation to toe-off.

For each phase, the following metrics were calculated: Absolute Phase Duration: Expressed in milliseconds (ms); Stance Percentage: Relative contribution of each phase to total stance duration (%) and Frames Indices (Minimum and Maximum Frame): Discrete sequential frame numbers generated by the acquisition software at a uniform sampling frequency, where Frame 0 represents the onset of initial contact (ICP onset). Minimum Frame and Maximum Frame correspond to the exact start and end frames of each phase within the stance timeline.

### Statistical analysis

All variables were calculated using descriptive statistics. Where appropriate, continuous variables were displayed as mean and standard deviation, or as median and interquartile range. Categorical variables were presented as percentages and frequencies. For the comparisons between groups, Mann-Whitney *U* test was applied to non-normally distributed continuous variables, while Fisher’s exact test was used for categorical variables. A *p* < 0.05 was considered statistically significant.

## Results

### Sociodemographic and descriptive data

Demographic and anthropometric characteristics of the 82 participants (41 cases, 41 controls) are summarized in Table 1. Both groups were matched for age, sex ratio, and foot length. Statistically significant differences were observed for Body Weight (59.76 ± 12.25 kg in cases vs. 66.49 ± 10.18 kg in controls; p < 0.001) and Body Mass Index (BMI) (21.56  ± 3.36 kg/m^2^ in cases vs. 24.08 ± 3.04 kg/m^2^ in controls; p < 0.001).

**Table 1:** Main characteristics of total sample with or without bilateral cavus foot.

	Total sample (n = 82)	Case Group (n = 41)	Control Group (n = 41)	
Factors	Mean ± SD (Range)	Mean ± SD (Range)	Mean ± SD (Range)	P-Value*
Age (years)	25.51 ± 5.99 (20 - 46)	26.07 ± 7.16 (20 - 46)	24.95 ± 4.55 (21 - 39)	0.940†
Weight (kg)	63.12 ± 11.69 (45 - 92)	59.76 ± 12.25 (45- 92)	66.49 ± 10.18 (53 - 89)	< 0.001†
Height (cm)	166.50 ± 0.87 (150 - 192)	166.93 ± 0.93 (150 - 192)	166.07 ± 0.83 (150 - 185)	0.780†
BMI (kg/m^2^)	22.82 ± 3.43 (16.65 - 32.44)	21.56 ± 3.36 (16.65 - 30.74)	24.08 ± 3.04 (19.00 - 32.44)	< 0.001†
Sex, male/female (%)	11/71 (13.4/86.6)	7/34 (17.1/82.9)	4/37 (9.8/90.2)	0.519 ‡
Foot Size (cm)	38.97 ± 2.50 (35 - 47.5)	38.82 ± 2.50 (35 - 47.5)	39.12 ± 2.52 (36 - 46)	0.414†

**Abbreviations:** kg, Kilogram; cm, Centimetre; %, Percentage; SD, Standard Deviation; n, Number.

† Mann-Whitney *U* test was used.

‡ Fisher exact test was used. In all the analyses,

*p < .05 (with a 95% confidence interval) was considered statistically significant.

### Primary outcome measures

The spatiotemporal metrics for both lower extremities are detailed in Table 2 that shows in detail the findings of the 24 variables tested divided into right and left foot, along with control and case group. It can be seen statistically significant differences (*p* < 0.05) when comparing the results of the case group with the control group in the following spatiotemporal characteristics: higher left foot FFCP percentage, lower left foot FFCP frame data, shorter left foot FFP time and percentage, and lower left foot FFP max frame values. The right foot of the case group had shorter FFP time, lower FFP percentage, higher ICP duration, ICP percentage, and ICP max frame data.

**Table 2:** Main outcome measurements of gait analysis of total sample with or without bilateral cavus foot.

	Total sample (n = 82)	Case Group (n = 41)	Control Group (n = 41)	
Factors	Mean ± SD (Range)	Mean ± SD (Range)	Mean ± SD (Range)	P-Value*
Left foot FFCP (ms)	244.30 ± 57.26 (148–414)	250.22 ± 45.92 (178–374)	238.39 ± 66.76 (148–414)	0.229 †
Left foot FFCP (%)	34.88 ± 8.31 (20–61)	37.05 ± 8.28 (24–61)	32.71 ± 7.85 (20–48)	0.020 †
Left foot Min Frame FFCP	48.99 ± 9.48 (23–70)	46.27 ± 10.09 (23–69)	51.71 ± 8.08 (34–70)	0.002†
Left foot Max Frame FFCP	73.56 ± 9.09 (39–94)	71.22 ± 9.97 (39–94)	75.90 ± 7.53 (58–89)	0.006 †
Left foot FFP (ms)	373.67 ± 101.24 (58–593)	341.61 ± 115.79 (58–554)	405.73 ± 72.40 (275–593)	0.002 †
Left foot FFP (%)	50.61 ± 11.34 (10 - 69)	47.17 ± 13.12 (10 - 67)	54.05 ± 8.00 (38 - 69)	0.026†
Left foot Min Frame FFP	11.00 ± 4.59 (2–28)	11.54 ± 5.69 (2–28)	10.46 ± 3.11 (3–17)	0.560 †
Left foot Max Frame FFP	49.00 ± 9.50 (23–70)	46.29 ± 10.12 (23–69)	51.71 ± 8.08 (34–70)	0.002 †
Left foot ICP (ms)	107.66 ± 45.07 (19–273)	112.83 ± 55.82 (19–273)	102.49 ± 30.70 (29–167)	0.527 †
Left foot ICP (%)	14.51 ± 6.67 (2–29)	15.78 ± 8.52 (2–39)	13.24 ± 3.77 (4–21)	0.397 †
Left foot Min Frame ICP	0 ± 0.00 (0–0)	0 ± 0.00 (0–0)	0 ± 0.00 (0–0)	1.000 †
Left foot Max Frame ICP	11.10 ± 4.65 (2–28)	11.54 ± 5.69 (2–28)	10.66 ± 3.32 (3–17)	0.678 †
Right foot FFCP (ms)	256.40 ± 100.28 (158–728)	270.56 ± 107.41 (158–728)	242.24 ± 91.75 (158–455)	0.085†
Right foot FFCP (%)	36.06 ± 12.63 (20–99)	38.34 ± 13.93 (23–99)	33.78 ± 10.88 (20–58)	0.121†
Right foot Min Frame FFCP	48.54 ± 12.73 (1–96)	46.51 ± 14.46 (1–96)	50.56 ± 10.53 (30–68)	0.102†
Right foot Max Frame FFCP	74.59 ± 11.41 (54–125)	74.00 ± 13.22 (55–125)	75.17 ± 9.40 (54–91)	0.138†
Right foot FFP (ms)	382.57 ± 116.13 (0–803)	347.95 ± 127.31 (0–803)	417.20 ± 92.98 (254–561)	< 0.001†
Right foot FFP (%)	51.85 ± 13.00 (0 - 84)	47.68 ± 14.05 (0 - 84)	56.02 ± 10.45 (34 - 70)	0.002 †
Right foot Min Frame FFP	9.66 ± 5.38 (1–29)	11.17 ± 6.41 (1–29)	8.15 ± 3.58 (1–14)	0.030 †
Right foot Max Frame FFP	48.55 ± 12.72 (1–96)	46.54 ± 14.45 (1–96)	50.56 ± 10.53 (30–68)	0.105 †
Right foot ICP (ms)	94.50 ± 52.98 (9.00–285)	109.29 ± 63.10 (9.00–285)	79.71 ± 35.35 (9.00–138)	0.034 †
Right foot ICP (%)	12.33 ± 6.97 (1–36)	14.46 ± 8.27 (1–36)	10.20 ± 4.55 (1–17)	0.024 †
Right foot Min Frame ICP	0 ± 0.00 (0–0)	0 ± 0.00 (0–0)	0 ± 0.00 (0–0)	1.000 †
Right foot Max Frame ICP	9.66 ± 5.38 (1–29)	11.17 ± 6.41 (1–29)	8.15 ± 3.58 (1–14)	0.030 †

**Abbreviations:** ICP, Initial Contact Phase; FFCP, Forefoot Contact Phase; FFP, Flatfoot Phase. ms, Phase Duration; %, Percentage; SD, Standard Deviation; N, Number.

† Mann-Whitney *U* test was used. In all the analyses,

*P < .05 (with a 95% confidence interval) was considered statistically significant.

For the right foot, the case group showed a significantly longer ICP duration (109.29 ± 63.10 ms vs. 79.71 ± 35.35 ms, p = 0.034), a higher ICP percentage (14.46 ± 8.27% vs. 10.20 ± 4.55%, p = 0.024), and an increased ICP Max Frame (11.17 ± 6.41 vs. 8.15 ± 3.58, p = 0.030). Concurrently, the right foot FFP duration was markedly reduced in the case group (347.95 ± 127.31 ms vs. 417.20 ± 92.98 ms, p < 0.001), as was the FFP percentage (47.68 ± 14.05% vs. 56.02 ± 10.45%, p = 0.002).

For the left foot, FFP duration was significantly shorter in the case group (341.61 ± 115.79 ms vs. 405.73 ± 72.40 ms, p = 0.002), with a reduced FFP stance percentage (47.17 ± 13.12% vs. 54.05 ± 8.00%, p = 0.026) and lower FFP Max Frame values (46.29 ± 10.12 vs. 51.71 ± 8.08, p = 0.002). Furthermore, the left foot FFCP percentage was significantly elevated in the case group (37.05 ± 8.28% vs. 32.71 ± 7.85%, p = 0.020), accompanied by lower FFCP Min Frame (46.27 ± 10.09 vs. 51.71 ± 8.08, p = 0.002) and Max Frame indices (71.22 ± 9.97 vs. 75.90 ± 7.53, p = 0.006).

## Discussion

This study demonstrates that individuals with bilateral pes cavus exhibit distinct alterations in dynamic stance phase timing compared to healthy controls with normal foot posture. Our hypotheses were supported: participants with pes cavus showed a shortened Flatfoot Phase (FFP), an elongated Initial Contact Phase (ICP), and an earlier transition into the Forefoot Contact Phase (FFCP).

The reduction in FFP duration (in both absolute milliseconds and stance percentage) in both feet reflects structural midfoot rigidity. In a normal foot posture, midfoot flexible deformation during FFP acts as a primary attenuation mechanism for ground reaction forces [23,24].

In cavus feet, joint stiffness and reduced plantar contact area limit midfoot stance duration, forcing an accelerated progression toward forefoot loading [5,23]. This temporal compression of the midfoot phase deprives the lower limb of efficient shock absorption, which may explain the higher incidence of foot instability and overload injuries reported in this population [13].

Significant differences were observed in this investigation’s findings during forefoot contact phase (FFCP) and flatfoot phase (FFP) between subjects with PC and with normal feet, the left foot FFCP was remarkably higher in the PC group (37.05% ± 8.28) compared to the control group (32.72 ± 7.85, p = 0.020). This indicates that people with PC, due to the midfoot stiffness and reduced capacity to absorb impact forces, rely more on their front section of the foot while walking. Furthermore, the minimum and maximum frame values for the left foot FFCP were considerably different between both groups (p = 0.002 and p = 0.006 for PC and normal feet group, respectively) strengthening the concept of altered time and progression of stance phase in PC patients.

Moreover, in relation to the flatfoot phase (FFP), PC individuals showed it to be shorter when compared to control group. Left foot FFP duration was less in PC group than in the normal feet group (341.61 ms ± 115.79 *versus* 405.73 ms ± 72.40, p = 0.002, respectively), explaining that PC participants spend shorter time in total contact of the foot on the ground. As PC participants tend to spend less time on midfoot phase, some investigations found the relationship between reduced plantar surface with a more unstable gait pattern [5,23]. As for the right foot FFP, similar results were observed; PC group had a much shorter flatfoot phase (347.95 ms ± 127.31) than the control group (417.20 ms ± 92.98, p < 0.001), which adds to the information of the biomechanical impact of a cavus foot.

Ultimately, the initial contact phase (ICP) of the right foot had a longer time period in the PC group compared to the control group (109.29 ms ± 63.10 *versus* 79.71 ms ± 35.35, p = 0.034), indicating that PC subjects may take longer to balance and stabilize during the ICP, probably because of their impaired foot cushioning. In relation to the time spent on ICP, the PC group had a significantly higher percentage (14.46% ± 8.27), whilst control group spent 10.20% ± 4.55, p = 0.024. This indicates that PC patients may not find efficient load distribution when coming into contact with the ground.

Biomechanical analysis reveals that the significant reduction in absolute duration and stance percentage of the Flatfoot Phase (FFP) in both feet of the pes cavus group reflects a fundamental failure in adaptive foot deformation. Whereas a normal arch relies on dynamic midfoot contact to distribute impact forces, the soft tissue stiffness and structural rigidity inherent to bilateral pes cavus compromise this mechanism, forcing an accelerated progression toward the forefoot. This reduction in FFP duration essentially deprives the gait cycle of its primary shock-attenuation mechanism. As a result, the early onset and extended duration of the Forefoot Contact Phase (FFCP) concentrate high magnitude mechanical stress on the metatarsal region. Consequently, these baropodometric alterations reinforce the clinical necessity for targeted orthotics that provide artificial midfoot support to alleviate forefoot overload.

Beyond localized foot pathologies, these chronic baropodometric alterations carry significant long-term consequences for the ascending kinetic chain. The structural rigidity and compromised dampening capacity of bilateral pes cavus prevent normal mechanical dissipation of ground reaction forces. Over time, these unmitigated impact forces are transmitted proximally, potentially contributing to accelerated micro-trauma and degenerative changes in the ankle, subtalar, and knee joints. Specifically, the lateral translation of mechanical stress associated with rigid high-arched mechanics underscores the importance of utilizing early dynamic podobarometric screening to mitigate long-term joint wear and musculoskeletal decline in this population.

These findings could be compared with other foot types and conditions that affect the dynamic function of the foot [25,26]. Existing literature suggests that the posture of the foot and its specific load distribution may contribute to the development of pathology in the ascending chain of the lower extremity [27,28]. Specifically, pressure management in the medial compartment of the knee is well-studied and correlated to flatfoot mechanics [27,29]. Most available and recent research describe knee overload, but do not focus on the implications of cavus foot and osteoarthritis of the lateral knee compartment, or lateral meniscus overload [28,30,31]. In this context, the obtained measurements may be useful as dynamic podobarometric biomechanical markers for future research on the upper ankle joints.

It is well known that PC leads to biomechanical difficulties, making early diagnosis and treatment crucial. A useful tool to image those pressure distribution abnormalities and for leading the way for futures interventions is de baropodometric platform [13]. The findings of the study highlight the increased forefoot pressure and lesser flatfoot phase duration compared to normal feet. Several studies suggest that redistributing plantar pressures is key to solving great number of PC problems, as metatarsalgia, callosities and ulcers [5]. For this purpose, insoles should be taken into consideration. From a clinical perspective, these spatiotemporal alterations highlight the importance of dynamic baropodometric evaluations beyond static structural measurements. Interventions such as custom-molded orthoses aimed at supporting the midfoot arch can prolong midfoot contact duration, reduce forefoot stress, and improve overall gait efficiency [8,9]. Complementary physical therapy programs focusing on intrinsic/extrinsic foot muscle conditioning and joint mobilization are also indicated [7,9].

This study has certain potential limitations, despite the valuable information provided. On the one hand, the participants were predominantly female, which may induce into mainly gender-based differences during gait dynamics and in foot structure of the PC itself; it would be advisable to opt for a more balanced sample of subjects between the two sexes. Additionally, while dynamic baropodometry provides accurate temporal stance data, incorporating kinematic motion capture [20,32], electromyography, and weightbearing radiography would offer comprehensive structural insights into cavus foot mechanics.

## Conclusions

Individuals with bilateral pes cavus demonstrate significant dynamic gait alterations characterized by prolonged initial heel contact duration, restricted flatfoot midfoot support, and a premature transition into forefoot contact. Dynamic baropodometric assessment provides functional spatiotemporal parameters that enhance clinical evaluation and help guide targeted conservative management in cavus foot deformities.
